# Early objective response to avelumab treatment is associated with improved overall survival in patients with metastatic Merkel cell carcinoma

**DOI:** 10.1007/s00262-018-02295-4

**Published:** 2019-02-05

**Authors:** Sandra P. D’Angelo, Matthias Hunger, Andrew S. Brohl, Paul Nghiem, Shailender Bhatia, Omid Hamid, Janice M. Mehnert, Patrick Terheyden, Kent C. Shih, Isaac Brownell, Céleste Lebbé, Karl D. Lewis, Gerald P. Linette, Michele Milella, Michael Schlichting, Meliessa H. Hennessy, Murtuza Bharmal

**Affiliations:** 1 Memorial Sloan Kettering Cancer Center, Weill Cornell Medical College, 300 East 66th Street, 12th Floor, New York, NY 10065 USA; 2 Mapi (An ICON plc Company), Munich, Germany; 3 Moffitt Cancer Center, Tampa, FL USA; 4 Division of Dermatology, Department of Medicine, Seattle Cancer Care Alliance, University of Washington, Seattle, WA USA; 5 Division of Medical Oncology, Department of Medicine, Seattle Cancer Care Alliance, University of Washington, Seattle, WA USA; 6 The Angeles Clinic and Research Institute, Los Angeles, CA USA; 7 Rutgers Cancer Institute of New Jersey, New Brunswick, NJ USA; 8 University of Lübeck, Lübeck, Germany; 9 Tennessee Oncology, Nashville, TN USA; 10 Center for Cancer Research, National Cancer Institute, Bethesda, MD USA; 11 Hôpital Saint-Louis, Paris, France; 12 University of Colorado Cancer Center, Anschutz, Aurora, CO USA; 13 Hospital of the University of Pennsylvania, Philadelphia, PA USA; 14 IRCCS Regina Elena National Cancer Institute, Rome, Italy; 15 Merck KGaA, Darmstadt, Germany; 16 EMD Serono, Billerica, MA USA

**Keywords:** Merkel cell carcinoma, Avelumab, PD-L1, Objective response, Overall survival, Endpoint validation

## Abstract

**Background:**

Response rates are primary endpoints in many oncology trials; however, correlation with overall survival (OS) is not uniform across cancer types, treatments, or lines of therapy. This study explored the association between objective response (OR) and OS in patients with chemotherapy-refractory metastatic Merkel cell carcinoma who received avelumab (anti-PD-L1).

**Methods:**

Eighty-eight patients enrolled in JAVELIN Merkel 200 (part A; NCT02155647) received i.v. avelumab 10 mg/kg every 2 weeks until confirmed progression, unacceptable toxicity, or withdrawal. Using conditional landmark analyses, we compared OS in patients with and without confirmed OR (RECIST v1.1). We applied a Cox model that included OR as a time-varying covariate and adjusted for age, visceral disease, and number of previous therapies.

**Results:**

Twenty-nine patients had confirmed OR; 20 by study week 7 and 7 more between study weeks 7 and 13. Survival probabilities 18 months after treatment initiation were 90% [95% confidence interval (CI) 65.6–97.4] in patients with OR at week 7 and 26.2% (95% CI 15.7–37.8) in patients without OR but who were alive at week 7. Median OS was not reached in patients with OR and was 8.8 months (95% CI 6.4–12.9) in patients without. Similar results were observed for the week 13 landmark. The adjusted Cox model showed OR was associated with a 95% risk reduction of death [hazard ratio 0.052 (95% CI 0.018–0.152)] compared with a nonresponse.

**Conclusions:**

Patients with OR by 7 or 13 weeks had significantly longer OS than patients without, confirming that early OR is an endpoint of major importance.

## Introduction

Merkel cell carcinoma (MCC) is a rare and aggressive neuroendocrine carcinoma of the skin [2–5]. Historically, survival rates in patients with MCC are poor, with an estimated mortality rate between 33 and 46% [6]. Metastatic MCC (mMCC) develops in approximately one-fifth of patients who present with local or regional disease [7].

Historically, the only treatment option in patients with mMCC was chemotherapy with a platinum agent. Despite the objective response rates (ORR) to chemotherapy as a first-line therapy for mMCC being relatively high, 52–61%, these responses were short-lived, with the median duration of response with first-line chemotherapy being approximately 3 months [8]. Although patients had objective responses (ORs) with chemotherapy, these responses did not translate into an overall survival (OS) benefit [6, 8–11]. In one study in patients presenting with mMCC, the 2-year survival rate was 11% [7].

Avelumab (MSB0010718C) is a fully human anti-PD-L1 IgG1 monoclonal antibody that inhibits interactions between PD-L1 and PD-1 but leaves intact the PD-L2/PD-1 pathway [12]. It is the first drug approved for the treatment of mMCC in a number of countries, including the United States and Japan, and the European Union. The approvals were based on data from the open-label, single-arm, multicenter clinical trial JAVELIN Merkel 200, which demonstrated a clinically meaningful and durable ORR [13, 14].

OS is considered the most reliable and clinically meaningful endpoint for evaluating drug efficacy in oncology clinical trials [15, 16]. However, evaluating OS requires large sample sizes and prolonged follow-up and can be confounded by postprogression therapies [17]. Thus, alternative endpoints, such as ORR and durable ORR, are being used as the primary endpoint in many oncology trials, yet these response rates have not been shown to correlate with OS across cancer types, treatments, or lines of therapy [17, 18].

Immunotherapy has been shown to improve OS compared with chemotherapy in various advanced cancer types, including melanoma and non-small cell lung cancer (NSCLC) [19, 20]. In particular, long-term clinical trial data show that a number of patients treated with checkpoint inhibitors experience a durable anti-tumor response [21], suggesting that the way patients’ disease responds to these treatments is different from their response to chemotherapy [22].

Previous studies in advanced NSCLC and renal cell carcinoma (RCC) have shown that in patients treated with anti-PD-1/PD-L1 antibodies, OR is associated with higher OS rates [22, 23]. In a recent meta-analysis of individual-patient-level data from 13 randomized immunotherapy trials of anti-PD-1/PD-L1 agents submitted to the US Food and Drug Administration (FDA), it was observed that patients with an OR had longer survival than patients whose disease did not respond [24].

Better understanding the relationship between OR and OS in patients treated with immunotherapy will help clinicians and decision makers assess therapeutic efficacy and potential for long-term clinical benefit.

The objective of this study is to investigate the association between OR and OS in patients with mMCC who were treated with avelumab.

## Materials and methods

### Study design

Data were analyzed from part A of trial EMR 100070-003/NCT02155647/JAVELIN Merkel 200, a single-arm, open-label, multicenter phase II study of avelumab as second-line or later therapy in patients with distant mMCC. Patients must have received ≥ 1 line of chemotherapy for the treatment of mMCC and had disease progression on or after the most recent line of chemotherapy. Eligible patients had an Eastern Cooperative Oncology Group performance status (ECOG PS) of 0 or 1 at trial entry, an estimated life expectancy of > 12 weeks, ≥ 1 unidimensional measurable lesion by RECIST v1.1, and adequate hematologic, hepatic, and renal function. Further study design details have been published previously [14].

Patients received avelumab at a dose of 10 mg/kg as a 1-h intravenous infusion every 2 weeks until confirmed disease progression, unacceptable toxicity, or occurrence of any other criterion for withdrawal. The date of data cutoff for the analyses was March 24, 2017, at which point all patients had ≥ 18 months of follow-up from the date of the last patient enrolled. Tumor assessment was performed every 6 weeks, and the radiological images by computed tomography or MRI and photographs of skin lesions were reviewed by an independent endpoint review committee (IERC) to determine response according to RECIST v1.1 [25].

OR [defined as either partial response (PR) or complete response (CR)] required confirmation of response per RECIST by IERC, preferably at the next regularly scheduled 6-week assessment and no sooner than 5 weeks.

### Statistical analyses

Comparative time-to-event analyses using time-dependent variables, such as tumor response, as predictors are different from analyses using baseline characteristics, which are fixed before the occurrence of any outcome event. This is because the covariate of interest (“response”) is changing with time: Patients whose disease responds to treatment must have at least survived from the time of treatment initiation to the time of response, whereas there is no such requirement for patients whose disease does not respond. To account for this “time-to-response” or “immortal time” bias, two approaches were used: the landmark analysis approach and extended Cox regression models with a time-varying covariate [26, 27].

In the landmark approach, a fixed time after the start of therapy is chosen as a landmark for analyzing survival by response. Only patients alive at the landmark are then included in the analysis and separated into two categories, distinguished by whether they experienced an OR up to that time. Consequently, the landmark method ignores all new ORs after the landmark and ignores all deaths before that time. OS is then analyzed conditional on the response status at the landmark time of patients who survived up to that time [27].

Two different landmarks were chosen: The first landmark was at week 7, covering responses experienced up to the first tumor assessment; the second landmark was at week 13 and covered responses that occurred between the first and second tumor response assessments. Survival probabilities in the two groups conditional on the response of patients at week 7 or week 13 were illustrated using Kaplan–Meier (KM) curves, and median OS was calculated in the two groups.

In contrast to the landmark method, the Cox regression model makes use of all patient data and does not ignore responses after a specific point in time [27]. The time-varying response variable included in the extended Cox regression model tracks whether the classifying event (“response”) has occurred during the estimation process. All patients would be classified initially as patients with nonresponse. Patients whose disease responded to treatment during follow-up would be switched into the responder group at the time that the response occurred and remain in that group until death. Different Cox regression models were applied. The first model included the time-varying covariate “response” as the only covariate. The second model additionally included age at baseline (continuous), the presence of visceral metastases (yes vs no) at baseline, and the number of previous therapies in the metastatic setting (1 vs > 1) as covariates. A third model additionally adjusted for PD-L1 expression, tumor Merkel cell polyomavirus status, ECOG PS, and tumor burden at baseline. Selection of these variables was based on clinical input, and these variables have been shown to be predictors of OS in patients with metastatic skin cancer [28].

Statistical analyses were performed using SAS v9.4 (SAS Institute, Inc; Cary, NC, USA).

## Results

### Patient population

In the 88 patients who received ≥ 1 dose of avelumab, median time since diagnosis of metastatic disease was 10.4 months (range 1.5–159.0 months; Table 1). Forty one percent of the patients had received ≥ 2 prior lines of chemotherapy, indicating a heavily pretreated population with advanced metastatic disease. The primary tumor site in most patients was skin. Confirmed ORs to avelumab were achieved in 29 patients [33.0%; 95% confidence interval (CI) 23.3–43.8%), 19 of whom had PR. Of the 29 patients with a confirmed response, 26 patients (KM estimate, 93%; 95% CI 75–98%) had a durable response of ≥ 6 months and 18 patients (KM estimate, 71%; 95% CI 51–85%) had a duration of response ≥ 12 months.

**Table 1:** Patient characteristics

Baseline characteristics	Patients (*N* = 88)
Median age (range), years	72.5 (33–88)
Age < 65 years, *n* (%)	22 (25)
Age ≥ 65 years, *n* (%)	66 (75)
Sex, *n* (%)	
Male	65 (74)
Female	23 (26)
Site of primary tumor, *n* (%)	
Skin	67 (76)
Lymph node	12 (14)
Other	2 (2)
Missing	7 (8)
Visceral disease at study entry, *n* (%)	
Present	47 (53)
Absent	41 (47)
ECOG PS, *n* (%)	
0	49 (56)
1	39 (44)
Merkel cell polyomavirus, *n* (%)	
Positive	46 (52)
Negative	31 (35)
Not evaluable	11 (13)
PD-L1 expression status, *n* (%)	
Positive	58 (66)
Negative	16 (18)
Not evaluable	14 (16)
Median sum of target lesion diameters at baseline per IERC (range), mm	79.0 (16–404) [*N* = 77]
Median time since first diagnosis (range), months	19.8 (2.9–159.0)
Median time since first diagnosis of metastatic disease (range), months	10.4 (1.5–159.0)
Previous systemic anticancer treatments, *n* (%)	
1	52 (59)
2	26 (30)
3	7 (8)
≥ 4	3 (3)
Follow-up and efficacy outcomes	
Median follow-up (range), months	23.0 (18.7–32.0)
Progression-free survival rate at 18 months (95% CI), %	29 (19–39)
OS rate at 18 months (95% CI), %	40 (29–50)
Confirmed best overall response, *n* (%)	
CR	10 (11)
PR	19 (22)
Stable disease	9 (10)
Progressive disease	32 (36)
Nonevaluable	18 (20)
Objective response rate (CR + PR) (95.9% CI), %^a^	33.0 (23.3–43.8)

*CI* confidence interval, *CR* complete response, *ECOG PS* Eastern Cooperative Oncology Group performance status, *IERC* Independent Endpoint Review Committee, *OS* overall survival, *PR* partial response

^a^Exact confidence interval using the Clopper–Pearson method

The proportion of patients who were progression free at 18 months was 29.0% (95% CI 19–39%). The estimated OS rate at 18 months was 40% (95% CI 29–50%) [29]. Nineteen patients (22%) received subsequent anticancer drug therapy.

### Landmark approach

Twenty patients were included in the group with response at week 7; 16 had a PR and 4 had CR prior to week 7. Five patients died, and 1 patient withdrew consent before week 7; these patients, all without OR, were not included in the week 7 landmark analysis. Twenty-seven patients were included in the response group at week 13; 22 had a PR and 5 had CR prior to week 13. Eleven patients died, and 2 patients withdrew consent before week 13; these patients, all without OR, were not included in the week 13 landmark analysis.

**Fig. 1:**
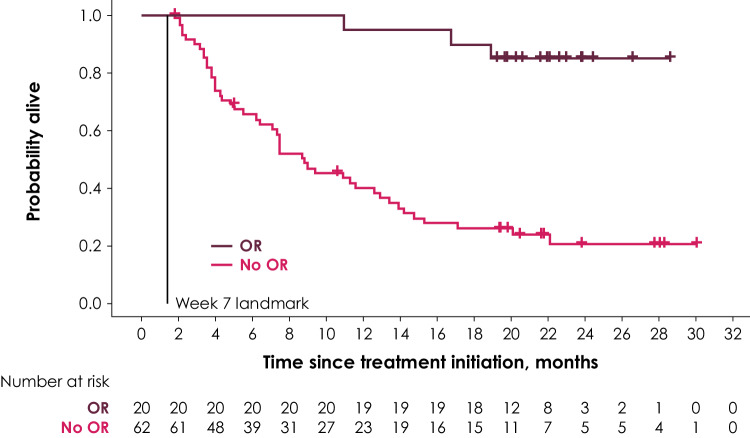
KM curve for OS by OR at the week 7 landmark

The KM curve for OS by tumor response at the week 7 landmark is shown in Fig. 1. Compared with the median OS of 8.8 months (95% CI 6.4–12.9 months) at week 7 in the group without response, the median OS at week 7 in the group with response was not reached. In the group without response at week 7, survival probabilities at 6, 12, and 18 months after treatment initiation (conditional on surviving week 7) were 65.5%, 40.1%, and 26.2%, respectively (Table 2). In the week 7 response group, survival probabilities at 6, 12, and 18 months after treatment initiation (conditional on surviving week 7) were 100%, 95.0%, and 90.0%, respectively (Table 2).

**Table 2:** Survival probabilities in response group and nonresponse group (conditional on surviving week 7 and week 13 landmarks)

Posttreatment initiation follow-up period	Week 7 landmark		Week 13 landmark	
	Patients with response (95% CI), %	Patients without response (95% CI), %	Patients with response (95% CI), %	Patients without response (95% CI), %
6 months	100 (100–100)	65.5 (52.1–76.0)	100.0 (100–100)	68.6 (53.4–79.8)
12 months	95.0 (69.5–99.3)	40.1 (27.7–52.2)	96.3 (76.5–99.5)	36.1 (22.7–49.7)
18 months	90.0 (65.6–97.4)	26.2 (15.7–37.8)	88.9 (69.4–96.3)	20.3 (10.1–33.0)

*CI* confidence interval

**Fig. 2:**
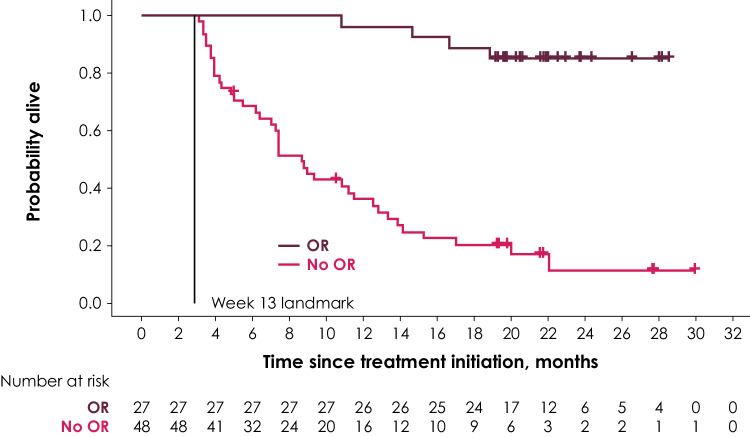
KM curve for OS by OR at the week 13 landmark

The KM curve for OS by tumor response at the week 13 landmark is shown in Fig. 2. Compared with the median OS of 8.7 months (95% CI 6.4–11.6 months) in patients without response at week 13, median OS in patients with response at week 13 was not reached. In general, there was a high similarity of estimated survival probabilities between the week 7 and week 13 landmark analyses, as reported in Table 2. Compared with the week 7 landmark analyses, conditional survival probabilities based on the week 13 landmark were slightly higher in the response group and slightly lower in the group without response at months 12 and 18.

Four patients had a CR before the week 7 landmark, of whom three were still alive at the date of data cutoff. One additional patient had a CR between the week 7 and week 13 landmarks but died at month 19.

### Cox regression model

In the unadjusted Cox regression model, any OR had a hazard ratio (HR) of 0.064 (95% CI 0.022–0.181), ie, an OR was associated with a 94% risk reduction of death, compared with nonresponse (Table 3). In the model adjusting for age, visceral metastases, and number of previous therapies, OR had a HR of 0.052 (95% CI 0.018–0.152) (Table 3). The HR for the presence of visceral metastases at baseline was 1.995 (95% CI 1.102–3.613), indicating that patients with visceral metastases had nearly twice the risk of death compared with patients without visceral disease. The association between OR and OS did not change when analyses were further adjusted for PD-L1 expression, Merkel cell polyomavirus status, ECOG PS, and tumor burden at baseline (results not shown).

**Table 3:** Regression output from Cox regression model with response as time-varying covariate

Parameter	Hazard ratio			*P* value
	Estimate	Lower limit 95% CI	Upper limit 95% CI	
Unadjusted model				
Response: OR vs no OR	0.064	0.022	0.181	< 0.0001
Adjusted model				
Response: OR vs no OR	0.052	0.018	0.152	< 0.0001
Age (per year)	1.011	0.986	1.036	0.3941
Visceral metastases at baseline: yes vs no	1.995	1.102	3.613	0.0226
Number of prior therapies: > 1 vs 1	1.037	0.596	1.802	0.8981

*CI* confidence interval, *OR* objective response

## Discussion

Clinical data from the JAVELIN Merkel 200 trial were used to investigate the association between tumor response and OS in patients with mMCC treated with avelumab. This is the first time the two outcomes have been tested for association in this indication, and results show that early OR (the majority of responses were PR and occurred by week 7) is a clinically relevant predictor of OS in patients with mMCC treated with second-line or later avelumab. This is important when placed into context of chemotherapy in mMCC, whereby responses can be very high, yet durability is dismal without an association with survival.

Results of the landmark analyses reveal considerably higher survival probabilities at 6, 12, and 18 months in patients with OR than in patients without a response. Having a response early, either at week 7 or week 13, is predictive of improved OS: 90% of these patients were still alive 18 months after treatment initiation, compared with 20–26% of patients without response at week 7 and 13. In addition, results from the Cox regression model showed that the association between OR (early or late) and OS remained stable when adjusted for patient characteristics that may impact survival; among those, only visceral metastases present at study baseline were associated with increased mortality.

Of the 48 patients without response at the week 13 landmark, 7 were still alive at the date of data cutoff (Fig. 2). Two of them had a late response after 18 and 36 months, respectively. The remaining 5 patients had good prognostic factors in that they all had no visceral disease at baseline and an ECOG PS of 0 as well as lower tumor burden at baseline, on average.

Increasing clinical experience indicates that traditional response criteria may not be sufficient to fully characterize response to immunotherapies [30, 31]. For example, mechanisms of action of immune-modulating agents are associated with delayed responses and flares in tumors associated with the influx of immune cells [32]. On these grounds, a tumor assessment system has been developed that incorporates these delayed or flare-type responses and designated immune-related response criteria (irRC) [33]. To supplement standard RECIST v1.1 evaluations, additional evaluations using modified irRC have been performed in this trial, combining the concepts of the irRC with RECIST v1.1. For modified irRC, only target and measurable lesions were considered, and ORRs in this trial were similar if modified irRC were used instead [14]. However, given the absence of definite modified criteria to be used in conjunction with immunotherapy, RECIST v1.1 continues to be used as the standard method to assess response to immunotherapy in clinical practice.

The strong association between OR and OS in patients with mMCC who are treated with second-line or later avelumab is important to clinical practice, in which this association could be used to make survival predictions earlier in a patient’s treatment. This is in contrast with chemotherapy, whereby responses can be very high; however, they are not durable, without association with survival. On the basis of these findings, physicians may be able to reassure patients whose disease demonstrates early response to avelumab that they may have an improved prognosis in terms of expected duration of survival. The observed ORR and corresponding survival probabilities with second-line or later avelumab represents a therapeutic improvement compared with historical results with chemotherapy—including first-line treatment, which rarely produces durable response lasting 6 months in mMCC and is associated with a low 2-year survival rate [8]. Interestingly, within the same disease, two mechanisms of action can result in profoundly different associations between OR and OS. Whereas in patients treated with avelumab, an OR is predictive of improved OS, this is not the case for cytotoxic chemotherapy, the prior standard of care: Although > 50% of patients have a robust initial response to first-line chemotherapy at 2–3 months, 95% of patients will have had disease progression by 15 months, with very little effect of whether there was response at 2–3 months [8].

The predictive and prognostic value of baseline CD positivity at the invasive tumor margin has been evaluated in part A of JAVELIN Merkel 200, and a nonsignificant trend toward higher response rate and longer OS with higher tumor CD8^+^ levels was observed, but given the small sample, the presence of tumor responses across all evaluated subgroups cannot be described as predictive or prognostic [34]. Future analysis of CD8^+^ vs response and survival will continue to be evaluated in first-line avelumab treatment for mMCC.

Previous studies in patients treated with anti-PD-1/PD-L1 antibodies in different tumor types have evaluated the association between tumor response and OS, with results that are consistent with those from JAVELIN Merkel 200. Motzer et al. performed a landmark analysis to examine the correlation between OR and OS in patients with advanced RCC treated with nivolumab [23]. In patients with a response up to the month 4 landmark, OS rates at 12 and 18 months were higher than those in patients with stable or progressive disease [23]. The 18-month OS rate in patients with advanced RCC and OR to nivolumab was 89%. A correlation between response and OS was also reported in the everolimus arm of the trial, although with a smaller magnitude and based on a lower ORR [23]. Similarly, a landmark analysis in patients with advanced NSCLC treated with anti-PD-1/PD-L1 antibodies conducted by Shukuya et al. reported a longer median OS in patients who had a PR between weeks 5–9 than in patients with stable or progressive disease [22]. Mushti et al. recently conducted a meta-analysis using pooled data from 13 active-controlled immunotherapy trials of anti-PD-1/PD-L1 agents submitted to the FDA between 2014 and 2016 [24]. They found that in the immunotherapy arms, patients with an OR had longer survival than patients without tumor response. Patients with response in the immunotherapy arms also had longer OS than those with response to standard treatment in the active control arms [24]. The 18-month OS rate in patients with OR in the immunotherapy arm was approximately 86%. These data from trials in other diseases treated with anti-PD-1/PD-L1 support the association found in the JAVELIN Merkel 200 study that patients with an OR with avelumab also have longer survival than historical data show with chemotherapy, as demonstrated by the 18-month OS rate of 90%.

By evaluating individual-patient-level surrogacy between OR and OS, this study addresses one of the criteria for validating surrogate endpoints set out by Buyse et al. [35]. However, a limitation is that trial-level surrogacy, i.e., the association between treatment effects on the two endpoints, cannot be evaluated for avelumab, because this study did not include a randomized active comparator arm and currently there are no data on OR and OS available from other avelumab trials. Additionally, although this study offers the only prospective dataset in this rare disease, the relatively small sample size—88 immune-competent and chemotherapy-refractory patients—may limit the generalizability of results to the mMCC population regardless of immune status and prior treatment.

Two further limitations to the landmark analysis method should be noted. First, there is a risk of bias stemming from excluding the deaths that occurred prior the week 7 and week 13 landmarks [17]. However, the strong association between OR and OS was confirmed in the Cox regression analysis, which makes use of all data. Second, analyses of surrogate endpoints could lead to inaccurate assumptions about causal relationships with OS. As stated in previous systematic literature reviews, the generalizability of the OR and OS association identified in this study to other treatment types may, therefore, be limited [17].

Finally, OS in patients in this trial may be impacted by subsequent anticancer therapies, and this may also affect the association between OR and OS. Because the majority of patients with response had durable, ongoing responses, subsequent anticancer therapy was less frequent in these patients than in the group without response; among the 21 patients who received subsequent anticancer drug therapy, only 4 were in the avelumab response group.

Clinical trials are ongoing with avelumab in other indications, including gastric/gastro-esophageal junction cancer, head and neck cancer, Hodgkin lymphoma, melanoma, mesothelioma, NSCLC, ovarian cancer, RCC, and urothelial carcinoma. These trials may provide further data to evaluate response to and survival with avelumab therapy.

In conclusion, avelumab therapy had a clinically meaningful impact on survival in patients with previously treated mMCC whose tumors responded by week 7 or week 13 vs those whose tumors did not respond. Early OR to avelumab was associated with a clinically meaningful high 18-month OS rate of 90%, likely driven by the sustained durable responses, neither of which were previously reported in the chemotherapy literature for mMCC [14].

## Data Availability

The datasets used during and/or analyzed during the current study are available from the corresponding author on reasonable request.

## Abbreviations

CI: Confidence interval
CR: Complete response
ECOG PS: Eastern Cooperative Oncology Group performance status
FDA: Food and drug administration
HR: Hazard ratio
IERC: Independent endpoint review committee
irRC: Immune-related response criteria
KM: Kaplan–Meier
mMCC: Metastatic Merkel cell carcinoma
NSCLC: Non-small cell lung cancer
OR: Objective response
ORR: Objective response rate
OS: Overall survival
PR: Partial response
RCC: Renal cell carcinoma
